# New developments of metformin in the clinical cancer area

**DOI:** 10.18632/oncotarget.26418

**Published:** 2018-12-07

**Authors:** Lev M. Berstein

**Affiliations:** Laboratory of Oncoendocrinology, N.N. Petrov National Medical Research Center of Oncology, St. Petersburg, Russian Federation

**Keywords:** metformin, cancer, therapy, combinations, resistance

The history of antidiabetic biguanide metformin usage in clinical oncology is long and interesting, even instructive. A brief but important, paper by Scottish researchers from the University of Dundee [1] is usually considered to be its starting point. In this retrospective analysis of outcomes in a cohort of diabetic patients, the authors noted a 23% lower cancer incidence (no data on tumor type were provided) in metformin recipients, thereby implying a metformin’s ability to reduce cancer risk in a time- and cumulative dose-dependent manner. Many of further studies have also focused on metformin use in cancer patients with diabetes. There have been a number of comprehensive reviews summing up this experience. For instance, the one by Heckman-Stoddard et al. [2] pinpointed some observational epidemiology data obtained in diabetic patients’ cohorts and underlined the importance of tumor site and the need for randomized studies [2]. This notion, although mentioned by previous authors [3], was implemented in the clinical cancer arena – due to different reasons - rather slowly. Nevertheless, the same paper by Heckman-Stoddard et al. [2] contained an ample reference to the MA.32 trial by National Cancer Institute of Canada (NCIC), a Phase III adjuvant breast cancer trial including more than 3500 breast cancer patients who received chemotherapy and irradiation during first year after diagnosis. These patients were randomized to receive metformin (850 mg twice daily) or placebo. The primary endpoint is a 5-year invasive disease-free survival (ClinicalTrial.gov registration no. NCT01101438, PI Prof. Pamela Goodwin [4]). This trial (whose results are awaited for 2020 or later) is notable not only due to its cohort size and randomized nature, but also due to the enrollment of *non-diabetic* women. The issue of the metformin’s (and other biguanides) potential chemopreventive and therapeutic properties in non-diabetic cancer patients has been a point of public interest and debate for several years (see, e.g. [5]) due, in no small part, to the influence of several publications authored by prof. Vladimir Dilman and his collaborators [6, 7, 8].

Metformin may have other potential clinical applications in combination with other standard or innovative cancer treatment modalities. While it may be added to adjuvant or neoadjuvant chemo- and hormone therapy, there is also relatively recent data on its potential combination with immune checkpoint inhibitors [9] and targeted therapy. Regarding the latter, the data from the Javier Menendez group recently published in *Oncotarget* by Martin-Castillo et al. [10] may serve as a good example here. This second phase trial studied a cohort of diabetes-free, early HER2-positive breast cancer patients receiving neoadjuvant metformin in combination with trastuzumab (a monoclonal antibody leading to HER2 downregulation) and chemotherapy. The patients were randomized into two groups. The first group received metformin (850 mg twice daily for 24 weeks) combined with 12 cycles of paclitaxel and trastuzumab followed by 4 cycles of 5-fluorouracil, epirubicin and cyclophosphamide. The second one received the same therapy regimen without metformin. According to the data obtained, there was no significant difference in pCR rate (65.5% for the first and 58.6% for the second group, *p* = 0.589), which was the primary end-point of the trial. There was, however, a tendency to higher organ-sparing surgery availability in metformin recipients compared to the other group (79.3% and 58.6%, accordingly; *p* = 0.089). Unfortunately, the authors failed to enroll a planned number of patients and, therefore, it is unclear whether the lack of significant difference in the study settings reflects a true lack of metformin efficacy. Since the triple combination employed (i.e., metformin plus chemotherapy plus targeted therapy) was well tolerated and safe, there is no doubt that a role for metformin in the clinical cancer arena merits further investigation [10].

What conclusions can we draw from all this? Here we should repeat, once more, that the story of metformin in clinical oncology is both interesting and instructive. Why so? The study by Martin-Castillo et al. [10] shows us a long and winding road covered by different obstacles, which stretched even before metformin can make its on way to clinical oncology. However, the shift from one line of analysis (patients with diabetes) to another (diabetes-free patients), and then to a third one (metformin combinations with different anti-cancer treatment modalities) may point the way to more effective use of this drug or toward better refinement of its current treatment concept. While moving in this direction, we should not forget that drug resistance might influence metformin response. Metformin’s effect markers may be metabolic (at the systemic level) and pharmacogenetic, while some other approaches may exploit also metabolic traits of tumor tissues (that is, at the local level). For example, it has recently been proposed to distinguish two groups of breast carcinomas in terms of their response to metformin-based neoadjuvant courses, namely: those with an increase in oxidative phosphorylation/OXPHOS gene transcription and tumor proliferation and those with increased 18-FDG uptake without change in proliferative activity [11]. Altogether, these tests may be useful clinical implications in metformin-containing regiments of treatment and prevention for different cancers.
